# Multiplex Bioanalytical Methods for Comprehensive Characterization and Quantification of the Unique Complementarity-Determining-Region Deamidation of MEDI7247, an Anti-ASCT2 Pyrrolobenzodiazepine Antibody–Drug Conjugate

**DOI:** 10.3390/antib12040066

**Published:** 2023-10-17

**Authors:** Yue Huang, Jiaqi Yuan, Ruipeng Mu, Robert J. Kubiak, Kathryn Ball, Mingyan Cao, G. Patrick Hussmann, Niluka de Mel, Dengfeng Liu, Lorin K. Roskos, Meina Liang, Anton I. Rosenbaum

**Affiliations:** 1 Integrated Bioanalysis, Clinical Pharmacology & Safety Sciences, R&D, AstraZeneca, 121 Oyster Point Boulevard, South San Francisco, CA 94080, USA; yue.huang@astrazeneca.com (Y.H.); jiaqi.yuan1@astrazeneca.com (J.Y.); rmu@arcusbio.com (R.M.); meina.liang@amadorbio.com (M.L.); 2 Clinical Pharmacology and Quantitative Pharmacology, Clinical Pharmacology & Safety Sciences, R&D, AstraZeneca, One MedImmune Way, Gaithersburg, MD 20878, USA; robert.kubiak@astrazeneca.com (R.J.K.); lorin.roskos@amadorbio.com (L.K.R.); 3 Clinical Pharmacology and Quantitative Pharmacology, Clinical Pharmacology & Safety Sciences, R&D, AstraZeneca, Granta Park, Cambridge CB21 6GH, UK; kathryn.ball@astrazeneca.com; 4 Department of Analytical Sciences, Biopharmaceutical Development, R&D, AstraZeneca, One MedImmune Way, Gaithersburg, MD 20878, USA; mingyan.x.cao@gsk.com (M.C.); patrick.hussmann@astrazeneca.com (G.P.H.); niluka.demel@astrazeneca.com (N.d.M.); dengfeng.liu.work@gmail.com (D.L.)

**Keywords:** deamidation, hybrid liquid chromatography–mass spectrometry (LC-MS) assay, post-translational modification (PTM), biotransformation, antibody–drug conjugate (ADC)

## Abstract

Deamidation, a common post-translational modification, may impact multiple physiochemical properties of a therapeutic protein. MEDI7247, a pyrrolobenzodiazepine (PBD) antibody–drug conjugate (ADC), contains a unique deamidation site, N102, located within the complementarity-determining region (CDR), impacting the affinity of MEDI7247 to its target. Therefore, it was necessary to monitor MEDI7247 deamidation status in vivo. Due to the low dose, a sensitive absolute quantification method using immunocapture coupled with liquid chromatography–tandem mass spectrometry (LBA-LC-MS/MS) was developed and qualified. We characterized the isomerization via Electron-Activated Dissociation (EAD), revealing that deamidation resulted in iso-aspartic acid. The absolute quantification of deamidation requires careful assay optimization in order not to perturb the balance of the deamidated and nondeamidated forms. Moreover, the selection of capture reagents essential for the correct quantitative assessment of deamidation was evaluated. The final assay was qualified with 50 ng/mL LLOQ for ADC for total and nondeamidated antibody quantification, with qualitative monitoring of the deamidated antibody. The impact of deamidation on the pharmacokinetic characteristics of MEDI7247 from clinical trial NCT03106428 was analyzed, revealing a gradual reduction in the nondeamidated form of MEDI7247 in vivo. Careful quantitative biotransformation analyses of complex biotherapeutic conjugates help us understand changes in product PTMs after administration, thus providing a more complete view of in vivo pharmacology.

## 1. Introduction

**Figure 1:**
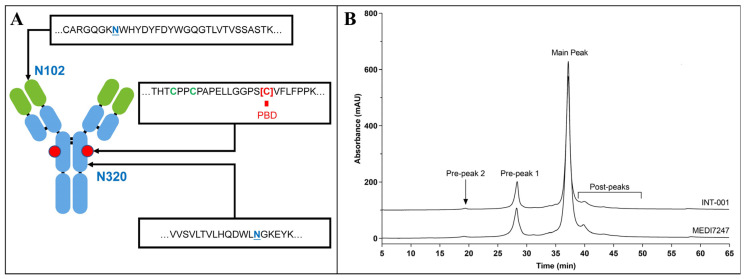
(**A**) MEDI7247 structure, with the N102, conjugation site and N320 enlarged; (**B**) ion exchange chromatography for the antibody (INT-001) and MEDI7247 of a representative lot, with the main peak and the two pre-peaks as labeled.

The neutral amino acid transporter ASCT2 belongs to solute carrier family 1 (SLC1) [1]. Although the acronym ASCT2 stands for alanine–serine–cysteine transporter 2, it has been found that the preferred substrate for ASCT2 is in fact glutamine [2]. Glutamine is a non-essential amino acid that can be synthesized from glucose [3]. Cancer cells display a high rate of glutamine uptake, often known as “glutamine addiction” [4]. ASCT2 was found to be frequently overexpressed in a variety of cancer cell lines [5], while normal tissues from vital organs appear to have restricted ASCT2 expression [6]. The broad upregulated expression of ASCT2 on cancer cells and its limited expression on normal cells have made it an attractive target for cancer therapy [7]. MEDI7247 is a site-specifically conjugated ADC (Figure 1A) with a drug-to-antibody ratio (DAR) of 2. MEDI7247 selectively binds the extracellular region of ASCT2, while sparing other members of the SLC1 family (e.g., ASCT1) [8]. MEDI7247 has demonstrated in vitro cytotoxicity potency [9] and was evaluated in Phase 1 clinical trials in selected relapsed/refractory hematological malignancies (NCT03106428) [6,10] and advanced or metastatic solid tumors (NCT03811652) [11]. Detailed characterization of MEDI7247 post-translational modification (PTM) found that MEDI7247 can undergo unique asparagine deamidation located at N102.

Deamidation is a common post-translational modification (PTM) for proteins. It can happen to asparagine or glutamine residues, changing the amino acid to aspartic acid or iso-aspartic acid, or glutamic acid or iso-glutamic acid, respectively [12]. A higher storage temperature or basic pH may increase the level of deamidation in vitro [13]. The kinetics of deamidation can be correlated with the surrounding amino acid sequence [14] and may be predictable [15,16,17]. Endogenous IgG also undergoes a deamidation process in vivo, e.g., at certain locations with asparagine (N) in the fragment crystallizable region (Fc region) [18]. For drug candidates, deamidation may affect the physiochemical properties and has often been evaluated as a critical quality attribute (CQA) in vitro or in vivo [18,19,20,21,22]. In MEDI7247, the deamidation N102 site located in the complementarity-determining region (CDR) affected the binding of MEDI7247 to ASCT2. MEDI7247’s mechanism of action relies on binding to the ASCT2 cell-surface receptor and subsequent internalization to deliver the cytotoxic payload. Therefore, N102 deamidation affects the cytotoxic potency of MEDI7247 and can further impact the pharmacology of MEDI7247. To address the impact of this deamidation on the pharmacokinetics (PK) and the efficacy of MEDI7247 in vivo, it was necessary to monitor deamidation from patient plasma samples. The quantitative in vivo monitoring of post-translational modifications is an important approach relating changes in PTMs to pharmacology of biotherapeutics. The quantification of in vivo deamidation changes poses significant analytical challenges, including the selection of the proper capture reagent as well as enzyme and signature peptide selection [22,23]. Previous pioneering work on deamidation analysis in vivo largely focused on evaluations of monoclonal antibodies (mAbs). In 2005, Huang et al. reported the relative quantification of deamidation from an IgG1 mAb [24]. Later, multiple manuscripts from Bults et al. reported the absolute quantification of in vivo deamidation [25], and connected it to mAb pharmacology [26]. Compared to mAbs, the monitoring of deamidation for PBD ADCs is inherently challenging as they are typically studied at very low clinical doses [27], hence requiring high-sensitivity assays to assess deamidation at clinically relevant concentrations.

Bioanalytical strategies for complex bioconjugates such as ADC typically rely on the measurement of three species (total antibody, conjugated payload (ADC) and released payload) and have been reviewed previously [28]. Herein, we report a sensitive absolute quantification method for the total and nondeamidated species of MEDI7247, as well as qualitative monitoring of the deamidated MEDI7247 in human plasma. The method used an anti-idiotope antibody (anti-ID) that was not affected by the deamidation of N102 as the capture reagent. A peptide specific to the deamidation site, a peptide that was not affected by the deamidation and the payload were monitored. The sample preparation conditions were fine-tuned to avoid increases in deamidation. Moreover, further investigation into deamidation using electron-activated dissociation (EAD) fragmentation confirmed iso-aspartic acid formation at a protein deamidated site. The multiplex LBA-LC-MS/MS method was qualified for the quantification of the MEDI7247 ADC, total MEDI7247 antibody and nondeamidated MEDI7247 antibody from a 50–5000 ng/mL human plasma concentration. The qualified method was then applied to a Diffuse Large B-Cell Lymphoma (DBLCL) cohort from clinical trial NCT03106428 [6]. To our knowledge, this is the first time an assay of such sensitivity was reported for the monitoring of in vivo deamidation in human plasma, informing our quantitative understanding of product PTM changes in vivo and their impact on ADC pharmacology.

## 2. Material and Methods

### 2.1. Materials

The MEDI7247 reference standard material (Ref) and the purified main peak (MP) and pre-peak (PP) materials, and the capture reagents, including two anti-idiotope antibodies (anti-IDs) and one anti-PBD antibody (anti-PBD), were provided by AstraZeneca (Gaithersburg, MD, USA). The peptides were synthesized at New England Peptide (Gardner, MA, USA). SMART IA beads, EZ-Link^®^ Sulfo-NHS-LC-Biotin, a Zeba™ Desalt Spin Column, acetonitrile (ACN) and formic acid (FA) were obtained from Thermo Scientific (Waltham, MA, USA). The enzymes, including IdeS, trypsin and chymotrypsin, were from Promega (Madison, WI, USA). The Rapigest, Acquity BEH C18 columns and HLB solid-phase extraction (SPE) plates were purchased from Waters (Middleton, MA, USA). LCMS-grade water was purchased from JT Baker. Protein LoBind vials and plates (Eppendorf, Hamburg, Germany) were used for all LCMS sample preparation. General reagents, buffers and plates were purchased from VWR (Radnor, PA, USA). Selected samples from patients participating in clinical study NCT03106428 were analyzed using the method described. This study was conducted in accordance with principles of the Declaration of Helsinki and the International Conference on Harmonization Guidance for Good Clinical Practice. Independent ethics committee approval was obtained.

### 2.2. Capture Reagent Selection

The ability of the capture reagents to bind deamidated forms of MEDI7247 was evaluated via the enzyme-linked immunosorbent assay (ELISA) method. Clear Nunc Maxisorp plates were coated overnight at 5 °C with 100 μL/well of 1 μg/mL solutions of the anti-MEDI7247 clones m1H10.1, r5A11.3 and 2G7-A8 in 0.2 M carbonate/bicarbonate. After overnight incubation at 5 °C, plates were washed with 0.1% Tween 20/PBS (4 times 300 μL/well) and blocked with 150 μL/well of 5% BSA in PBS for approximately 1 h at room temperature. MEDI7247 reference standard materials (main peak and pre-peaks, with different levels of deamidation) were used to prepare calibration standards in assay buffer (0.5% BSA, 0.1% Tween 20 in PBS) at concentrations ranging from 128 ng/mL to 0.125 ng/mL. Calibration standards were added into the blocked and washed plates and incubated for approximately 1 h at room temperature. Plates were washed as described above and incubated for 1 h at room temperature at a 1:12,500 dilution in assay buffer of goat anti-human antibody conjugated to horseradish peroxidase (Bethyl Laboratories, Montgomery, TX, USA). After the washing step, 100 μL/well of TMB (SeraCare, Milford, MA, USA) was added to each plate and incubated for approximately 10 min before stopping with 100 μL/well of 0.2 M sulfuric acid. Plates were read using a SpectraMax340 PC plate reader (Molecular Devices, San Jose, CA, USA) at 450 nm with background subtraction at 650 nm.

### 2.3. Reference Material Characterization

The reference materials for MEDI7247 deamidation were prepared in PBS (pH = 7.4) at 0.1 μg/mL. A 20 μL aliquot was digested first with IdeS (37 °C, 1 h), and then, mixed with 0.1% Rapigest and 0.1 M dithiothreitol (DTT) for denaturation and reduction (60 °C, 0.5 h). The samples were then cooled to ambient temperature and alkylated with excess iodoacetamide (IAM) (in the dark, 1 h). Subsequently, samples were buffer-exchanged to 80 μL PBS and digested with trypsin or chymotrypsin (1:20 enzyme-to-protein ratio) at 37 °C overnight. The samples were acidified with 10 μL of 1% (*v*/*v*) FA before injecting 20 μL for analysis.

The digested reference materials were analyzed via the ion-dependent acquisition method (IDA) using an SCIEX 6600 TOF (Framingham, MA, USA). The digested samples were loaded onto a BEH C18 column (PN. 186002352) from Waters (Milford, MA, USA) with 2% mobile phase A (0.1% FA in water), and then, separated with a gradient of 2 to 10% mobile phase B (0.1% (*v*/*v*) FA in acetonitrile) within 1 min, followed by 10 to 70% mobile phase B in 35 min. For every full scan, 6 precursors were fragmented. The data were first analyzed using BioPharmaView Version 3.0 from SCIEX to search for potential PTMs and miscleavages, followed by manual verification. Then, the peak areas of the key deamidation sites were extracted via MultiQuant™ Version 3.0.3863 software (SCIEX) using ±50 ppm of the accurate mass for the observed charge states within m/z 200–1600.

### 2.4. Method Qualification and Sample Testing

Biotinylated anti-ID m1H10.1 was generated by incubating the antibody with an EZ-Link^®^ Sulfo-NHS-LC-Biotin water solution followed by purification. The product was then incubated with streptavidin SMART IA beads at a ratio of 4 μg protein to 30 μL beads. The beads were washed thoroughly and reconstituted to the original volume prior to use. Immuno-capture was performed by incubating 50 μL human plasma and 30 μL of previously prepared SMART IA beads at 37 °C with constant shaking for 2 h. After incubation, the bead mixture was washed multiple times. Then, a SMART Digestion IA buffer with internal standards spiked was added to each well, followed by tryptic digestion at 70 °C for 2 h with constant shaking. After digestion, 4/5 of the sample volume was transferred to a Waters HLB solid-phase extraction (SPE) plate. Samples in the SPE cartridge were first washed with water and 5% acetonitrile, and then, eluted with 30% methanol in acetonitrile. The remaining 1/5 of the sample volume was incubated overnight at room temperature with excessive papain to release the conjugated payload. The papain-digested sample was also treated using the SPE plate in a similar manner, except for the elution condition (10% methanol in acetonitrile). In both cases, the elution was further diluted in 0.1% FA in water before injection to the LCMS. An Agilent Bravo™ automated liquid handling system was used for plasma sample preparation, including the preparation of calibration standards and quality controls, bead washing, SPE purification, etc. Shimadzu Nexera ultra-high-performance liquid chromatography (UHPLC) coupled with an SCIEX 6500+ system was used for method qualification and sample analysis. The separation was performed on a Waters BEH C18 column (product number: 186002350) with a gradient separation of 0.1% FA in water and 0.1% FA in acetonitrile, respectively (Table S2). Data were acquired using Analyst™ software (Analyst 1.7 with HotFix 3). Peak area integration and linear regression were performed with MultiQuant™ software (Version 3.0.3863).

### 2.5. Pharmacokinetic Parameter Calculation

Individual plasma concentration–time data were used to estimate individual pharmacokinetic (PK) parameters using noncompartmental analysis (NCA) in Phoenix^®^ WinNonlin^®^ version 6.4 (Certara USA, Inc., Princeton, NJ, USA). The nominal administered dose was used for each patient, and the actual sampling times were used. Pre-dose data that were below the limit of quantitation (BLQ) were set to zero, and then, for each patient and each analyte, the first BLQ sample after the maximum observed plasma concentration (Cmax) was set to half of the lower limit of quantitation (LLOQ), and any subsequent BLQ samples in the profile were set to zero. The area under the plasma concentration–time curve (AUC) was calculated using the linear-up log-down method. Plasma clearance (CLplasma), volume of distribution at steady-state (Vss) and terminal elimination half-life (T1/2,z) were estimated using the AUC extrapolated to infinity, with the terminal elimination rate constant estimated using the ‘best fit’ calculation method. Summary statistics (arithmetic mean and standard deviation) were calculated for each PK parameter. Graphical presentation of plasma concentration–time profiles was carried out in R [29] using the package ggplot2 [30], using nominal sampling times, and all BLQ data set to half-LLOQ.

### 2.6. Characterization of Iso-Aspartic Acid Reference Material via EAD

Anti-human IgG-Fc was conjugated to SMART Digest ImmunoAffinity (IA) Streptavidin beads. MEDI7247 reference material (60 µg) was diluted with SN1 buffer (1 mg/mL BSA in 50 mM Tris, pH 7.5) to reach a 50 µg/mL final concentration before incubation with 1200 µL conjugated beads (2 h, 25 °C). The beads were then washed thoroughly with 50 mM Tris buffer, pH 7.5, before being redispersed with SMART Digest ImmunoAffinity (IA) digestion buffer (3.6 mL). After 2 h digestion at 70 °C, the supernatant was loaded onto a Waters HLB SPE plate and washed sequentially with water and 5% methanol. Analytes were then eluted with methanol. The eluate was concentrated down to approximately 80 µL using a Thermo Scientific Savant SpeedVac vacuum concentrator. The remaining eluate was mixed at a 1:1 ratio (*v*:*v*) with 0.1% FA in water, before injection into the LCMS. A Shimadzu ExionLC coupled with a SCIEX ZenoTOF 7600 System was used for data acquisition. The separation was performed using a Waters BEH C18 column (product number: 186002350) with a gradient separation of 0.1% FA in water and 0.1% FA in acetonitrile, respectively. The LC gradient was set with a mobile phase B increase from 1 to 10% at 5 min to 25 min, and then, 10 to 30% at 25 min to 65 min, followed by additional washing. EAD fragmentation was performed using a TOF MS/MS method using a 7 eV electron KE, 0.6 s accumulation time, 5500 V spray voltage, 10 V CE, 5000 nA electron beam current and 30 ms EAD reaction time. Data were acquired and analyzed using SCIEX OS™ software (Version 3.1.5.3945).

## 3. Results

**Figure 2:**
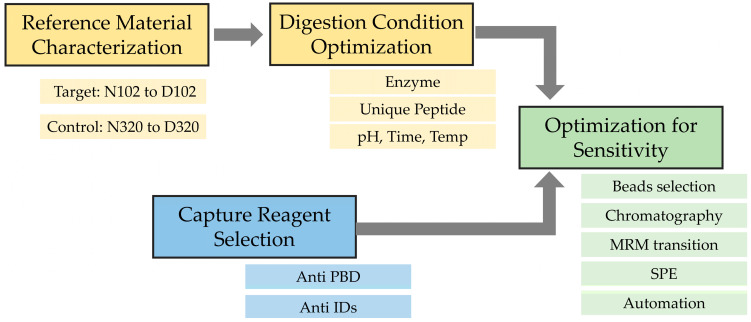
Method development flowchart for the monitoring of MEDI7247 in vivo deamidation assay.

To develop a sensitive bioanalysis assay in the human matrix for in vivo PTM monitoring, it is critical to ensure that the immunocapture and the LCMS monitoring of the target analyte is specific and selective and the sample process condition does not introduce additional PTMs or quantitatively change their abundance. Therefore, key assay elements such as the reference standard, digestion condition and capture reagents need to be individually evaluated during method development, and then, act in concert for optimal performance to reach the required assay sensitivity (Figure 2). From the ion exchange chromatography (IEC) of MEDI7247 and the mAb before conjugation, two major pre-peaks associated with the deamidation products were observed (Figure 1B). The pre-peak and main peak materials were collected as fractions and purified. In this study, four different materials were used for the method development and characterization of the impact of deamidation on the quantification of MEDI7247: (1) the main peak material (MP); (2) pre-peak 1 (PP1), the pre-peak that eluted later and was closer to the main peak; (3) pre-peak 2 (PP2); and (4) the MEDI7247 reference standard (Ref) that was not separated, with approximately 20–25% of the total pre-peaks in the IEC. The DARs for materials 1–4 were comparable, with the main difference being the CDR deamidation.

### 3.1. Characterization of Reference Material

**Figure 3:**
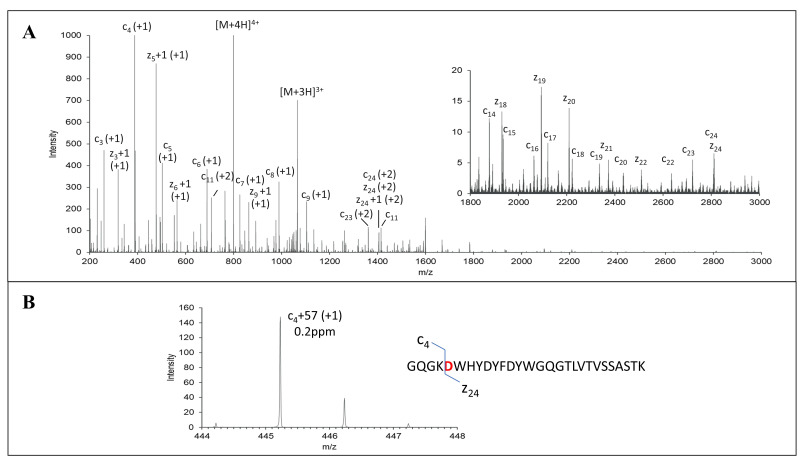
EAD MS^2^ spectra of deamidated peptide of MEDI7247 trypsin digestion sample. (**A**) EAD MS^2^ mass spectrum of 200–3000 m/z. Zoomed-in spectrum of 1800–3000 m/z is shown as an insert. (**B**) EAD MS^2^ spectrum of c_4_+57 signature ion of deamidation peptide. Sequence and cleavages are noted on the insets.

The initial characterization of MEDI7247 revealed that the heavy-chain CDR region N102 had the highest deamidation rate. It was also observed that the trypsin cleavage at the preceding lysine was affected. Due to the possibility of aspartic acid or iso-aspartic acid formation during N102 deamidation in MEDI7247, the EAD MS/MS spectrum was acquired to determine the deamidation product. The signature fragment ions c+57 and z-57 were only observed in iso-aspartic acid MS/MS fragmentation [31], and therefore, can be used to differentiate between aspartic acid and iso-aspartic acid. MEDI7247 (PP1) tryptic digestion was analyzed using reverse-phase LC coupled to SCIEX 7600 ZenoTOF, and the only deamidated peptide peak was observed at a retention time 43.6 min. After EAD fragmentation, a series of c and z ions were observed, including iso-aspartic acid signature ions c_4_+57 (Figure 3B) and z_24_-57 (Figure S1), confirming that iso-aspartic acid is the major deamidation product.

### 3.2. Impact of Deamidation on MEDI7247 Capture with Anti-IDs and Anti-PBD Antibody

**Figure 4:**
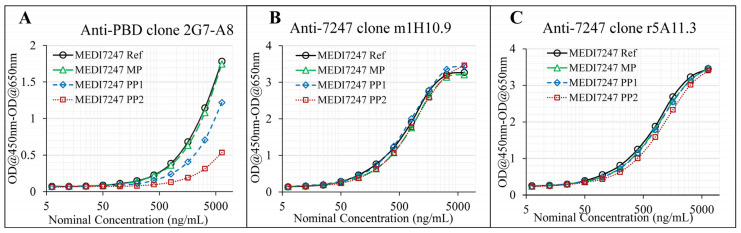
Evaluation of the capture reagents. (**A**) anti-PBD antibody shows significant difference in capture capability for the pre-peaks, compared with the main peak; (**B**) anti-ID m1H10.9 and (**C**) anti-ID r5A11.3 were not affected by the deamidation of the reference material.

Three capture reagents, including two anti-IDs against the CDR region and one anti-PBD payload antibody, were examined for their capture ability for MEDI7247. The impact of deamidation on the binding of these three capture reagents was evaluated using MP, PP1, PP2 and Ref. Both anti-IDs’ ability to bind MEDI7247 (m1H10.9 and r5A11.3) was not impacted by N102 deamidation (Figure 4B,C). For anti-PBD 2G7A8, reduced binding to MEDI7247 was observed with PP1 and PP2 material (Figure 4A). Since the DARs of MP, PP1 and PP2 are comparable, the change in anti-PBD antibody binding characteristics suggests a potential tertiary structure change from the unique iso-aspartic acid deamidation. For the development of this method, anti-ID m1H10.9 was selected as the capture antibody.

### 3.3. Enzyme Selection and Unique Peptide Selection

**Figure 5:**
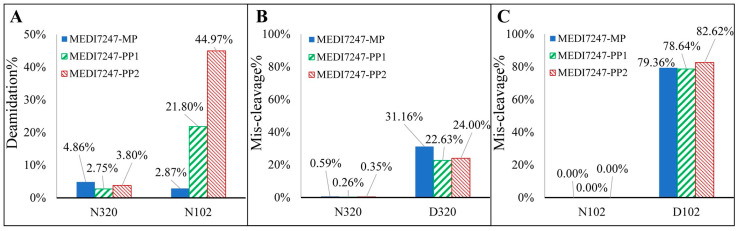
(**A**) The low deamidation % of N320 indicates that little deamidation was introduced during sample preparation, while the deamidation % of the N102 in the main peak, pre-peak 1 and pre-peak 2 increased. (**B**) The miscleavage % of the N320 peptide is below 1%, while that of the D320 peptide is around 25%; (**C**) the miscleavage% of the N102 peptide is undetectable, while that of the D102 peptide results in ~80% for the miscleavage peptide.

From the characterization of the reference standards, deamidation at multiple locations other than N102 was observed. Among the additional deamidation sites, the majority showed less than a 1% deamidation rate, with the exception of N320, located in the heavy-chain fragment constant region (Fc). Since N320 appeared to be a sensitive site to deamidation, it was monitored together with N102 during assay optimization as a control. For bioanalytical assays monitoring deamidation, the evaluation of key aspects of the sample preparation procedure, such as the high-pH buffer, time of incubation and heating, is essential as spontaneous deamidation may accelerate during sample preparation and introduce bias [13]. Therefore, it is also important to evaluate miscleavage, which could result in incomplete digestion and thus affect quantification, even under well-defined gentle conditions aimed at reducing in-process deamidation. It has also been noted that enzymatic digestion efficiency may differ between the original and the deamidated peptides. Multiple peptides from N102 and N320 were monitored to evaluate the digestion efficiency and potential in-process deamidation, including the deamidated and nondeamidated peptides as well as the miscleavage peptides (Table 1). For all the peptides, the observed charge states and retention times are listed. From the results, miscleavage was observed for both chymotrypsin and trypsin. However, much higher miscleavage rates at non-specific digestion sites were also observed in chymotrypsin digestion. Therefore, trypsin was selected for digestion and the tryptic peptides were further evaluated (Figure 5). The peak area for the listed peptides were acquired by summing all the extracted ion chromatograms (XIC) from z = 1 to 5, with ±50 ppm of the accurate mass. Based on the XIC peak area (PA) at the designated retention time, the deamidation% and misleavage% were calculated using Equations (1) and (2), respectively.

(1) $$deamidation\%=\frac{total\;Peak\;Area\;of\;the\;deamidated\;peptides}{total\;Peak\;Area\;of\;the\;non-deamidated\;and\;deamidated\;peptide}$$

(2) $$miscleavage\%=\frac{total\;Peak\;Area\;of\;the\;miscleavage\;peptides}{total\;Peak\;Area\;of\;the\;peptide\;and\;miscleavage\;peptides}$$

**Figure 6:**
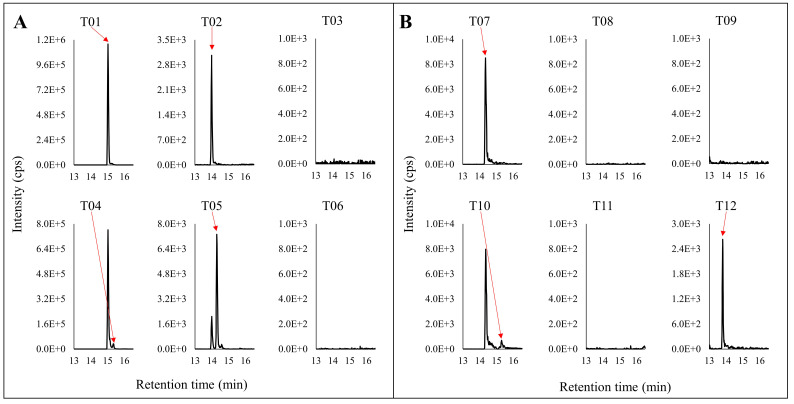
Representative chromatograms of the tryptic digestion peptides for (**A**) N320 and (**B**) N102. The top panel is nondeamidated peptides. The lower panel is the deamidated version of the corresponding top panel peptides.The red arrows point to the specific peak in the chromatograms corresponding with the label.

There was limited N320 deamidation observed in all three MEDI7247 materials, indicating that little deamidation was induced during the sample preparation procedure (Figure 5A). It is also obvious that in the MP material, little deamidation was observed on N102, while in PP1 and PP2, the deamidation % increased. It was expected that the deamidated peptide would have a different digestion efficiency compared to the original. The N320 peptide showed little miscleavage, while D320 showed consistently around 20–30% miscleavage (Figure 5B). However, N102 and D102 had a large difference in their miscleavage percentages (Figure 5C). While N102 had no observed miscleavage, D102 largely favored the miscleavage peptide, with a consistent miscleavage rate of 80%. An example of the chromatogram for the 12 tryptic peptides from Table 1 can be found in Figure 6. Selected Multiple Reaction Monitoring (MRM) for each peptide with low background interference was used (Table S1). The digestion conditions evaluated were then repeated with modified capture steps and similar results were observed.

### 3.4. Method Qualification

From the aforementioned evaluations, the selected capture reagent, magnetic beads and target peptides were combined to build the method using a SCIEX triple-quadrupole (QTRAP) 6500+ system for qualification. A well-characterized material (Ref) with about a 24% total pre-peak was used as the reference standard. Usually, for absolute quantification, an authentic reference standard of acceptable purity is required. Since it was not feasible to use a fully deamidated MEDI7247 as the reference material, a 24% total pre-peak material was selected as the reference standard, which has a very close pre-peak percentage to that of the dosing material. With this reference standard, the proportion of nondeamidated antibody among the total antibody was quantifiable. The deamidated peptide T12 was also monitored, but only qualitatively. Due to the lack of reference standard for fully deamidated material and uncertainty in digestion efficiency, direct absolute quantification of deamidation via monitoring of the peptide containing D102 was not possible; instead, we relied on the quantification of the nondeamidated form for the quantitative assessment. In addition to the nondeamidated and deamidated signature peptides, the total antibody concentration was monitored using a CDR peptide that was not affected by deamidation and the ADC concentration was measured by releasing the payload using papain following tryptic digestion. This method was qualified via thorough examination of its accuracy, precision and selectivity following relevant regulatory guidance [32,33]. The accuracy and precision information for this multiplex method can be found in Table 2 and the selectivity results are listed in the Supplementary Materials (Table S3).

### 3.5. Clinical Deamidation Results

**Figure 7:**
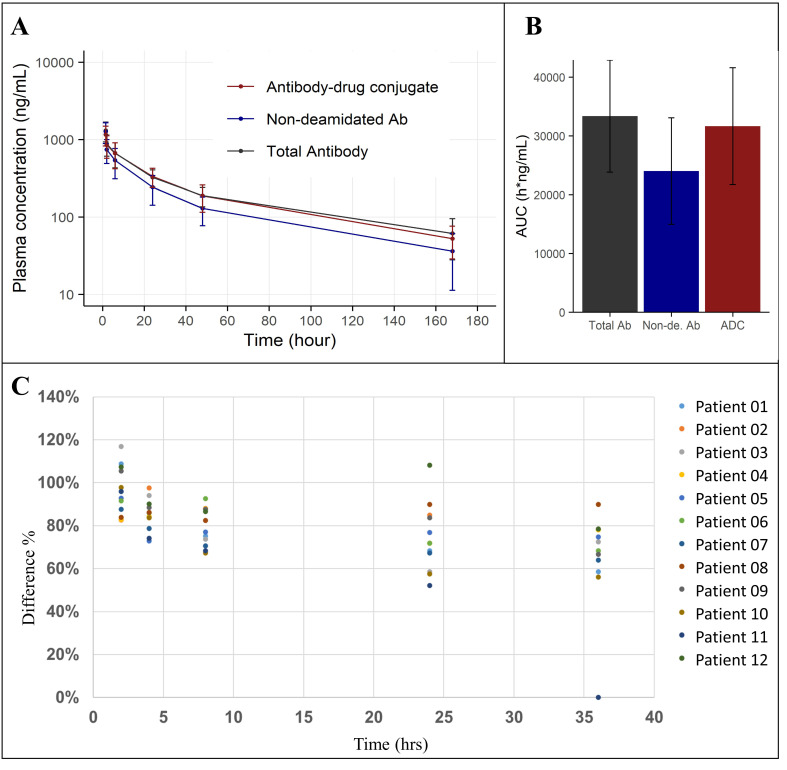
(**A**) Average plasma concentration–time plot of MEDI7247 ADC (red), nondeamidated Ab (blue) and total antibody (black) for eleven patients from one of the DLBCL cohorts; (**B**) the AUC_0-last_ (h × g/mL) for total antibody, nondeamidated antibody and ADC; (**C**) the percentage of nondeamidated Ab in total Ab decreasing with time.

MEDI7247 was evaluated in clinical trial NCT03106428 to treat patients with selected relapsed/refractory hematological malignancies, including Diffuse Large B-cell Lymphoma (DLBCL). Selected samples from one of the DLBCL patient cohorts were tested using this qualified method. The mean plasma concentration–time profile from 11 patients dosed with MEDI7247 is shown in Figure 7A. The AUCs for the three measurements can be found in Figure 7B, and the details for each individual patient in Table S4. In addition to the total Ab and ADC concentrations, the degree of deamidation in vivo was also quantified. This could be achieved by comparing the quantification results from the nondeamidated peptide T07 with the total Ab and ADC or by monitoring the changes in T12. To evaluate the deamidation process over time, the concentrations of nondeamidated Ab and total Ab were compared using Equation (3) (Figure 7C). Since the reference standard and the dosing material have similar degrees of deamidation, this effectively represents the initial deamidation status in vivo. This was observed at the early time points. However, the nondeamidated Ab% showed increasing negative bias at later time points (Figure 7C). This indicated a decrease in nondeamidated Ab among the total Ab in the circulation. Since minimal deconjugation of PBD was observed from the ADC and total Ab data (Figure 7), we can infer that the proportion of deamidated ADC within the total ADC follows a similar pattern to total Ab and deamidation, gradually increasing in the circulation.

(3) $$nondeamidated\;\%=\frac{\;\left[nondeamidated\;Ab\right]}{\left[total\;Ab\right]}$$

## 4. Discussion

Protein post-translational modification (PTM) is an inherent aspect of biotherapeutics. PTMs located in critical domains such as CDRs can alter binding to the receptor, which may further impact the biotherapeutic’s in vivo pharmacological properties. There are several approaches to connecting PTM to clinical pharmacological impact. First, proteins with certain PTMs can be separated, purified and evaluated using in vitro systems to evaluate changes in binding, activity or cytotoxicity. Bults et al. studied PTM using binding and in vitro cell viability assays [26]. For MEDI7247, the binding and in vitro cytotoxicity change for a PP1 material is approximately 5 times less compared to MP, while that for a PP2 material is approximately 10–20 fold less (manuscript in preparation). MEDI7247 deamidation at N102 has a clearly demonstrated impact based on in vitro evaluations. Secondly, while in vitro characterization can be helpful to understand the potential clinical impact, it is critical to initially assess the PK profiles of the ADC regardless of deamidation status. Subsequently, the impact of deamidation on PK can then be included in exposure–response relationship analysis. Therefore, a well-characterized, qualified assay with demonstrated accuracy and precision is also required for the PK characterization of MEDI7247. The interpretation of the results from the clinical sample testing in the context of assay performance and some inherent limitations is critical to evaluating the potential impact of the unique N102 CDR PTM.

The development of the LBA-LCMS assay addressing MEDI7247 deamidation presented a number of unique challenges. To match the clinically relevant concentrations of ADCs, a sensitive LBA-LCMS assay was needed. First, a well-characterized reference material is essential for absolute quantification assays, especially for bioconjugates with PTM, where heterogeneity and biotransformation may further complicate the characterization and quantification of target analytes. For MEDI7247, while the iso-aspartic acid deamidation product was confirmed using a synthetic internal standard peptide, EAD offers another orthogonal method to verify iso-aspartic acid formation (Figure 3). The second key aspect for the development of bioanalytical assays in the human matrix is to ensure selectivity and specificity at the required sensitivity, which can only be achieved with a combination of a capture reagent, a well selected enzyme and a signature peptide. Here, several selective antibodies against MEDI7247 were evaluated. Interestingly, although the deamidation site was located in CDR3, both anti-IDs targeting MEDI7247 CDR were not impacted by deamidation. On the contrary, the ability of the anti-PBD capture antibody to bind the payload, which was conjugated close to the hinge region, was impacted the most (Figure 4). One hypothesis explaining this phenomenon could be the tertiary structure change elicited by N102isoD deamidation. This result demonstrated the importance of the careful evaluation of capture reagents, especially for molecules with potential protein PTM or biotransformation in vivo. In this assay, trypsin was selected due to the highly specific digestion under well-controlled gentle digestion conditions. With iso-aspartic acid being the main deamidation product, a high level of miscleavage was observed (Figure 5). This resulted in a challenge to directly measure deamidated peptides, since all forms of miscleavage peptides with different ionization efficiency need to be monitored and controlled. Therefore, a more robust assay format using the nondeamidated peptide with a clean digestion profile was selected for quantification to avoid potential data ambiguity. The final assay format with nondeamidated antibody, total antibody and ADC quantification, demonstrated accuracy and precision and provided the deamidation status in vivo. The third key aspect is the detailed optimization of the assay performance, including MRM selection, chromatographic adjustments, SMART IA beads, the BRAVO automation platform and SPE. When selecting the appropriate MRM transition, in silico digestion and the protein Basic Local Alignment Search Tool (BLAST) identified transitions that had the potential to be highly selective for further evaluation. They were then further evaluated with selectivity samples, leading to the selection of the final MRM with the least interference from the background (Table S2).

This qualified method was applied to clinical samples, and enabled the monitoring of the total antibody and nondeamidated antibody in vivo. First, the time-averaged concentration profile data in Figure 7C show the gradual separation of nondeamidated MEDI247 and total MEDI7247 observed in the circulation. The possible causes for such an observation include the possibility that MEDI7247 further deamidated in vivo, or a difference in clearance rate between the different forms, e.g., the nondeamidated material cleared faster from the circulation, causing an apparent difference in the deamidation status. It is also possible that both causes described above contributed to the observed trend. Currently, we cannot exclude either possibility in contributing to this observation. It is possible that a larger study with more patients could address this question more definitively. It is important to analyze the changes in PTM in vivo while considering that the biological system can both form newly biotransformed species and can clear the dosed material or biotransformation products. Therefore, it is important to evaluate the overall impact from the calculated PK parameters. For MEDI7247, while the average clearance rate of the nondeamidated antibody (5.44 mL/h/kg, SD = 3.06) is slightly higher than that of the total antibody (3.44 mL/h/kg, SD = 1.56), it is not significant. Therefore, we cannot conclude that deamidation has a significant impact on MEDI7247 human PK in the patient population and at the dose level evaluated. Second, it is also worth noting that individual patients have different deamidation profiles (Figure S2). There are certain patients who showed almost no difference between the nondeamidated and total antibody concentrations (patients 03, 07 and 08) throughout the time monitored, while other profiles showed a clear difference (patients 01, 06, 09, 10 and 11). It is possible that diverse patient populations may respond differently to a certain PTM. This is an example of how individual patient monitoring is important for gaining a full understanding of PTM’s impact on PK and even efficacy. Outside the example of MEDI7247, if there is any demonstrated significant impact of PTM on PK, then it may be helpful to further characterize the relationship between patients’ demographic information and other biomarker information to relate the potential differences in PTM’s impact. Individual patient evaluation can help glean potential pharmacological impact and may assist in our understanding of the different covariates contributing to the complex biotherapeutic PK and efficacy.

## 5. Conclusions

In summary, protein PTMs may have an impact on the efficacy and immunogenicity profiles of therapeutic proteins [34,35,36]. This warrants further research on changes in therapeutic protein PTM in vivo. Here, a sensitive LBA-LC-MS/MS method was developed and qualified to monitor the absolute levels of the in vivo deamidation of MEDI7247 in patients. The method development and qualification procedure presented herein provides an example of how the deamidation of a biotherapeutic can be quantitatively monitored in vivo in the context of a clinical trial. Furthermore, we presented the application of a novel fragmentation technique, EAD, to the characterization of isomerization at the deamidation site. In quality sciences, PTMs are frequently a research priority for the characterization of biotherapeutics due to their potential impact on multiple functional attributes. In bioanalysis, when a major PTM site is located in critical regions such as the CDR, detailed quantification of the PTM in vivo helps to evaluate changes in the product’s critical quality attributes (CQA) after dosing to patients. This biotransformation analysis may inform future protein engineering efforts for new generations of drug candidates and can assist in risk assessments for this specific CQA and contribute to the quality control strategies for drug products.

## Figures and Tables

**Table 1:** The nondeamidated and deamidated peptides (0 and 1 miscleavage) from heavy-chain N102 and N320.

Index	Enzyme	Deamidation Site	Miscleavage	Sequence	Accurate Mass	Observed RT (min)	Observed Charge States
C01	Chymotrypsin	N320	0	NGKEY	609.2759	2.83	[M+H]+; [M+2H]2+
C02	Chymotrypsin	N320	1	NGKEYKCKVSNKAL	1580.8345	NA	NA
C03	Chymotrypsin	N320	1	LNGKEY	722.3600	4.36	[M+H]+; [M+2H]2+
C04	Chymotrypsin	D320	0	DGKEY	610.2599	4.03	[M+H]+; [M+2H]2+
C05	Chymotrypsin	D320	1	DGKEYKCKVSNKAL	1581.8185	NA	NA
C06	Chymotrypsin	D320	1	LDGKEY	723.3440	4.49	[M+H]+; [M+2H]2+
C07	Chymotrypsin	N102	0	CARGQGKNW	1018.4767	4.18	[M+2H]2+; [M+3H]3+
C08	Chymotrypsin	N102	1	CARGQGKNWHY	1318.5990	NA	NA
C09	Chymotrypsin	N102	1	YCARGQGKNW	1181.5401	4.46	[M+3H]3+
C10	Chymotrypsin	D102	0	CARGQGKDW	1019.4572	4.32	[M+2H]2+; [M+3H]3+
C11	Chymotrypsin	D102	1	CARGQGKDWHY	1319.5794	NA	NA
C12	Chymotrypsin	D102	1	YCARGQGKDW	1182.5205	NA	NA
T01	Trypsin	N320	0	VVSVLTVLHQDWLNGK	1806.9993	15.04	[M+2H]2+; [M+3H]3+; [M+4H]4+
T02	Trypsin	N320	1	VVSVLTVLHQDWLNGKEYK	2227.2001	13.99	[M+3H]3+; [M+4H]4+
T03	Trypsin	N320	1	STSYNSTYRVVSVLTVLHQDWLNGK	2866.4614	NA	NA
T04	Trypsin	D320	0	VVSVLTVLHQDWLDGK	1807.9833	15.35	[M+2H]2+; [M+3H]3+
T05	Trypsin	D320	1	VVSVLTVLHQDWLDGKEYK	2228.1842	14.30	[M+3H]3+; [M+4H]4+
T06	Trypsin	D320	1	STSYNSTYRVVSVLTVLHQDWLDGK	2867.4454	NA	NA
T07	Trypsin	N102	0	NWHYDYFDYWGQGTLVTVSSASTK	2824.2770	14.35	[M+2H]2+; [M+3H]3+; [M+4H]4+
T08	Trypsin	N102	1	NWHYDYFDYWGQGTLVTVSSASTKGPSVFPLAPSSK	3991.9058	NA	NA
T09	Trypsin	N102	1	GQGKNWHYDYFDYWGQGTLVTVSSASTK	3194.4734	NA	NA
T10	Trypsin	D102	0	DWHYDYFDY WGQGTLVTVSSASTK	2825.2610	15.29	[M+2H]2+; [M+3H]3+; [M+4H]4+
T11	Trypsin	D102	1	DWHYDYFDYWGQGTLVTVSSASTKGPSVFPLAPSSK	3992.8898	NA	NA
T12	Trypsin	D102	1	GQGKDWHYDYFDYWGQGTLVTVSSASTK	3195.4574	13.80	[M+3H]3+; [M+4H]4+

**Table 2:** Qualification of the LCMS method measuring the MEDI7247 ADC, total Ab and nondeamidated antibody.

QC Levels	LLOQ	LQC	MQC	HQC	ULOQ	Dilutional QC (5 fold)
Nominal Concentration (ng/mL)	50	125	625	3750	5000	2500
MEDI7247 ADC	Released PBD Payload					
Average Recovery	116.8	94.9	100.7	106.8	101.0	109.7
CV%	18.1%	8.4%	9.9%	6.4%	8.9%	5.1%
Linear Regression	y = 2.79514e − 4x−0.00434 (weighting: 1/x); r = 0.99921					
Total Ab	Heavy-Chain CDR Unique Peptide: GLEWIGEIHHSGGANYNPSLK					
Average Recovery	120.0	88.5	109.6	109.4	112.2	117.3
CV%	21.4%	14.2%	13.1%	12.3%	2.1%	7.6%
Linear Regression	y = 2.39471e − 4x + 0.00490 (weighting: 1/x); r = 0.99696					
Nnondeamidated Ab	Nondeamidated Peptide: NWHYDYFDYWGQGTLVTVSSASTK					
Average Recovery	111.2	109.2	100.3	107.6	102.1	115.8
CV%	12.1%	11.2%	5.4%	5.1%	4.3%	7.3%
Linear Regression	y = 0.00103x − 0.02309 (weighting 1/x^2^); r = 0.99486					

## Data Availability

All relevant data required to replicate this study’s findings are within the paper and the Supplementary Materials. Raw data can be obtained in accordance with AstraZeneca’s data sharing policy, described at https://astrazenecagrouptrials.pharmacm.com/ST/Submission/Disclosure (accessed on 7 September 2023).

## Supplementary Materials

The following supporting information can be downloaded at: https://www.mdpi.com/article/10.3390/antib12040066/s1, Figure S1. EAD MS2 spectra of signature z24-57 ion of deamidation peptide of MEDI7247 trypsin digestion sample. (A) +2 charge ion and (B) +1 charge ion; Figure S2. Plasma concentration–time plot for 11 patients; Table S1: Mass spectrometer parameters for the (1) total Ab, (2) nondeamidated Ab and (3) MEDI7247 ADC assays. The deamidated antibody is qualitatively monitored; Table S2. Chromatographic gradient for the total Ab and nondeamidated Ab assays; Table S3. Selectivity of the multiplex assay with a spike in the LLOQs of 6 individual plasma lots; Table S4. Individual patient PK parameters calculated from ADC assay, nondeamidated (non-de.) Ab assay and total Ab assay.
