# Supplementary material for: Multiplex Bioanalytical Methods for Comprehensive Characterization and Quantification of the Unique Complementarity-Determining-Region Deamidation of MEDI7247, an Anti-ASCT2 Pyrrolobenzodiazepine Antibody–Drug Conjugate
Source: Antibodies (Basel). 2023 Oct 17;12(4):66. doi: 10.3390/antib12040066 (PMC10594446; doi:10.3390/antib12040066)

Supporting Information

Multiplex Bioanalytical Methods for Comprehensive Characterization and Quantification of the Unique Complementarity-Determining-Region Deamidation of MEDI7247, an Anti-ASCT2 Pyrrolobenzodiazepine Antibody–Drug Conjugate

Yue Huang [1], Jiaqi Yuan [1], Ruipeng Mu [1], Robert J. Kubiak [2], Kathryn Ball [3], Mingyan Cao [4], G. Patrick Hussmann [4], Niluka de Mel [4], Dengfeng Liu [4], Lorin K. Roskos [2], Meina Liang [1] and Anton I. Rosenbaum [1,*]

[1] Integrated Bioanalysis, Clinical Pharmacology & Safety Sciences, R&D, AstraZeneca, 121 Oyster Point Boulevard, South San Francisco, CA 94080, USA; yue.huang@astrazeneca.com (Y.H.); jiaqi.yuan1@astrazeneca.com (J.Y.); rmu@arcusbio.com (R.M.); meina.liang@amadorbio.com (M.L.)

[2] Clinical Pharmacology and Quantitative Pharmacology, Clinical Pharmacology & Safety Sciences, R&D, AstraZeneca, One MedImmune Way, Gaithersburg, MD 20878, USA; robert.kubiak@astrazeneca.com (R.J.K.); lorin.roskos@amadorbio.com (L.K.R.)

[3] Clinical Pharmacology and Quantitative Pharmacology, Clinical Pharmacology & Safety Sciences, R&D, AstraZeneca, Granta Park, Cambridge, CB21 6GH, UK; kathryn.ball@astrazeneca.com

[4] Department of Analytical Sciences, Biopharmaceutical Development, R&D, AstraZeneca, One MedImmune Way, Gaithersburg, MD 20878, USA; mingyan.x.cao@gsk.com (M.C.); patrick.hussmann@astrazeneca.com (G.P.H.); niluka.demel@astrazeneca.com (N.d.M.); dengfeng.liu.work@gmail.com (D.L.)

* Correspondence: anton.rosenbaum@astrazeneca.com

Table S1. Mass spectrometer parameters for 1) the total Ab, 2) non-deamidated Ab and 3) MEDI7247 ADC assay. The deamidated antibody is qualitatively monitored.

Method Index	MRM Index	Analyte Name	Fragment Ion	Q1	Q3	dwell time (msec)	DP	CE	Used in Absolute Quantification
1 Total Ab	1	HC-CDR_GLEW	y18-2+	760.832	991.089	20	135	30	
	2		y16-2+	760.832	841.402	20	135	30	Y
	3	HC-CDR_GLEW_IS	y18-2+	763.499	995.089	20	135	30	
	4		y16-2+	763.499	845.402	20	135	30	Y
2 Non-de. Ab	5	HC-CDR_NWHY	b9+	943.010	1305.347	30	135	42	Y
	6		y8+	943.010	780.853	30	135	42	
	7		y12+	943.010	1151.302	30	135	42	
	8	HC-CDR_NWHY_IS	b9+	945.677	1305.347	30	135	42	Y
	9		y8+	945.677	788.853	30	135	42	

	10		y12+	945.677	1159.302	30	135	42	
* De. Ab	11	HC-CDR_GQGK	y9+	800.356	879.985	30	135	35	
	12		y8+	800.356	780.853	30	135	35	
	13	HC-CDR_GQGK_IS	y9+	802.356	887.985	30	135	35	
	14		y8+	802.356	788.853	30	135	35	
3 ADC	15	SG3199	n/a	585.3	504.3	200	85	31	Y
	16	SG3199_IS	n/a	595.3	514.3	200	85	31	Y

MRM: multiple reaction monitoring; Q1: quadrupole 1 parent ion m/z; Q3: quadrupole 3 fragment ion m/z; DP: declustering potential; CE: collision energy; m/z: mass over charge ratio; HC: heavy chain; CDR: complementarity determining region; IS: internal standard.

Table S2. Chromatographic Gradient for the total Ab and non-deamidated Ab assay.

Time	MPB%
0.01	15
0.2	20
4	26.5
4.2	98
4.7	98
5	15
5.5	15

Table S3. Selectivity of the multiplex assay with the spiked in LLOQ of 6 individual plasma lots.

	Individual 1	Individual 2	Individual 3	Individual 4	Individual 5	Individual 6
MEDI7247 ADC						
Rep1	84.43	79.98	150.51	97.77	79.94	105.91
Rep2	96.98	104.94	121.16	93.81	136.33	106.76
Rep3	98.39	104.81	133.42	114.89	104.93	115.11
pass/total	3/3	3/3	1/3	3/3	2/3	3/3
pass/fail	pass	pass	fail	pass	pass	pass
Total Ab						
Rep1	93.77	92.96	102.23	127.48	101.35	135.87
Rep2	102.01	88.13	91.5	119.9	112.4	118.97
Rep3	93.17	143.12	96.09	102.17	136.49	132.95
pass/total	3/3	2/3	3/3	2/3	2/3	1/3
pass/fail	pass	pass	pass	pass	pass	fail
Non-Deamidated Ab						
Rep1	102.94	86.23	93.85	138.34	83.25	98.2
Rep2	93.07	98.28	78.76	86.61	129.05	105.95
Rep3	125.06	97.73	89.43	112.84	115.7	104.5
pass/total	3/3	3/3	3/3	2/3	2/3	3/3
pass/fail	pass	pass	pass	pass	pass	pass

Table S4. Individual patient PK parameters calculated from ADC assay, non-deamidated (non-de.) Ab assay and total Ab assay.

Analyte	Patient ID	AUCinf h*ng/mL	AUClast h*ng/mL	CL mL/h/kg	Cmax ng/mL	Half-life h	Vss mL/kg
**ADC**	Patient 01	43700	38300	2.75	1150	56.0	199
	Patient 02	35500	29500	3.38	953	67.3	297
	Patient 03	44400	35900	2.70	1130	62.8	221
	Patient 04	26800	20200	4.47	669	65.9	421
	Patient 05	42800	36700	2.81	1280	72.5	245
	Patient 06	39100	34100	3.07	1450	59.6	224
	Patient 07	44300	38400	2.71	1090	59.3	207
	Patient 08	32600	30900	3.68	1400	47.0	204
	Patient 09	25100	23500	4.78	1040	43.2	250
	Patient 10	12700	12400	9.41	740	10.3	150
	Patient 11	49700	48300	2.42	1830	38.7	111
	**N**	**11**	**11**	**11**	**11**	**11**	**11**
	**Mean**	**36100**	**31700**	**3.84**	**1160**	**53.0**	**230**
	**SD**	**10900**	**9940**	**2.00**	**329**	**17.6**	**80.2**
	**CV%**	**30.3**	**31.4**	**52.2**	**28.4**	**33.2**	**34.9**
	**Median**	**39100**	**34100**	**3.07**	**1130**	**59.3**	**221**
	**Range**	**36900**	**35900**	**7.00**	**1160**	**62.2**	**310**
	**Geometric Mean**	**34000**	**29900**	**3.53**	**1110**	**48.2**	**218**

Analyte	Patient ID	AUCinf h*ng/mL	AUClast h*ng/mL	CL mL/h/kg	Cmax ng/mL	Half-life h	Vss mL/kg
**Non-de. Ab**	Patient 01	25100	23500	4.77	1320	44.9	239
	Patient 02	22400	20600	5.36	1230	50.3	314
	Patient 03	41300	32300	2.91	1260	69.8	258
	Patient 04	13200	11300	9.11	625	52.0	602
	Patient 05	29700	27700	4.05	1410	53.2	225
	Patient 06	26100	24400	4.59	1590	46.3	244
	Patient 07	42100	31600	2.85	993	87.3	322
	Patient 08	29600	27800	4.05	1480	49.2	225
	Patient 09	19200	17200	6.26	1040	54.9	397
	Patient 10	9330	8930	12.9	961	11.2	198
	Patient 11	40400	38800	2.97	2070	42.6	138
	**N**	**11**	**11**	**11**	**11**	**11**	**11**
	**Mean**	**27100**	**24000**	**5.44**	**1270**	**51.1**	**287**
	**SD**	**11000**	**9070**	**3.06**	**381**	**18.5**	**125**
	**CV%**	**40.6**	**37.8**	**56.2**	**30.0**	**36.1**	**43.4**
	**Median**	**26100**	**24400**	**4.59**	**1260**	**50.3**	**244**
	**Range**	**32800**	**29900**	**10.0**	**1440**	**76.1**	**464**
	**Geometric Mean**	**24700**	**22100**	**4.85**	**1220**	**46.7**	**268**

Analyte	Patient ID	AUCinf h*ng/mL	AUClast h*ng/mL	CL mL/h/kg	Cmax ng/mL	Half-life h	Vss mL/kg
**Total Ab**	Patient 01	42200	36400	2.84	1210	61.0	216
	Patient 02	28200	26800	4.25	1050	40.3	216
	Patient 03	52600	41100	2.28	1520	67.9	203
	Patient 04	15600	14000	7.69	674	43.5	433
	Patient 05	35900	34300	3.34	1540	45.4	171
	Patient 06	42100	37300	2.85	1810	58.2	201
	Patient 07	46300	35600	2.59	1180	83.5	276
	Patient 08	35400	33800	3.39	1400	43.9	180
	Patient 09	38900	30000	3.09	1060	81.5	326
	Patient 10	32700	26100	3.67	1000	68.0	393
	Patient 11	65800	51700	1.82	1930	93.2	207
	**N**	**11**	**11**	**11**	**11**	**11**	**11**
	**Mean**	**39600**	**33400**	**3.44**	**1310**	**62.4**	**257**
	**SD**	**13000**	**9530**	**1.56**	**373**	**18.2**	**89.3**
	**CV%**	**32.9**	**28.6**	**45.3**	**28.5**	**29.2**	**34.8**
	**Median**	**38900**	**34300**	**3.09**	**1210**	**61.0**	**216**
	**Range**	**50200**	**37700**	**5.86**	**1250**	**52.9**	**262**
	**Geometric Mean**	**37400**	**31900**	**3.21**	**1260**	**60.0**	**244**

N: number of animals; AUC_{inf}: area under the plasma concentration-time curve from start of dosing until infinity; AUC_{last}: area under the plasma concentration-time curve from start of dosing until time of last quantifiable concentration; CL: apparent plasma clearance; C_{max}: maximum observed plasma concentration; Half-life: terminal elimination half-life; V_{ss}: apparent volume of distribution at steady-state

Figure S1. EAD MS^2 spectra of signature z_{24}-57 ion of deamidation peptide of MEDI7247 trypsin digestion sample. A) +2 charge ion and B) +1 charge ion.

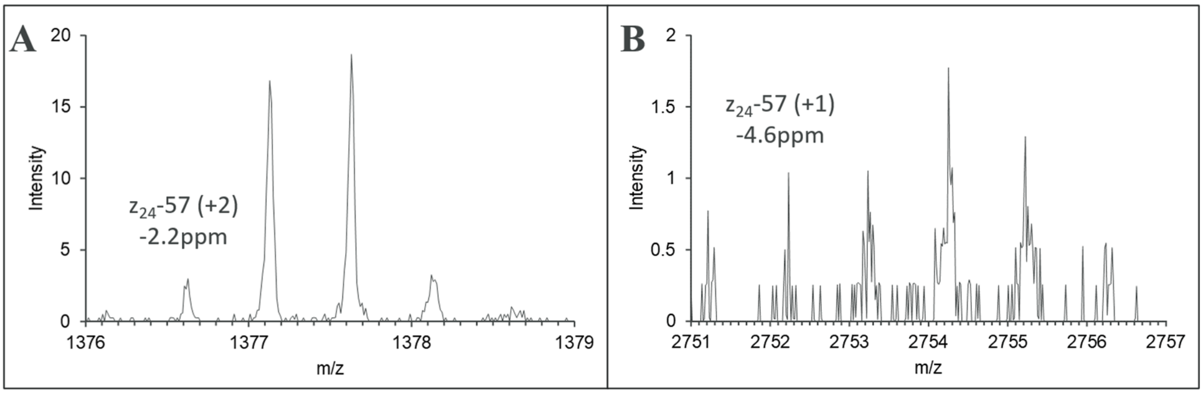

Figure S2. Plasma concentration-time plot for 11 patients

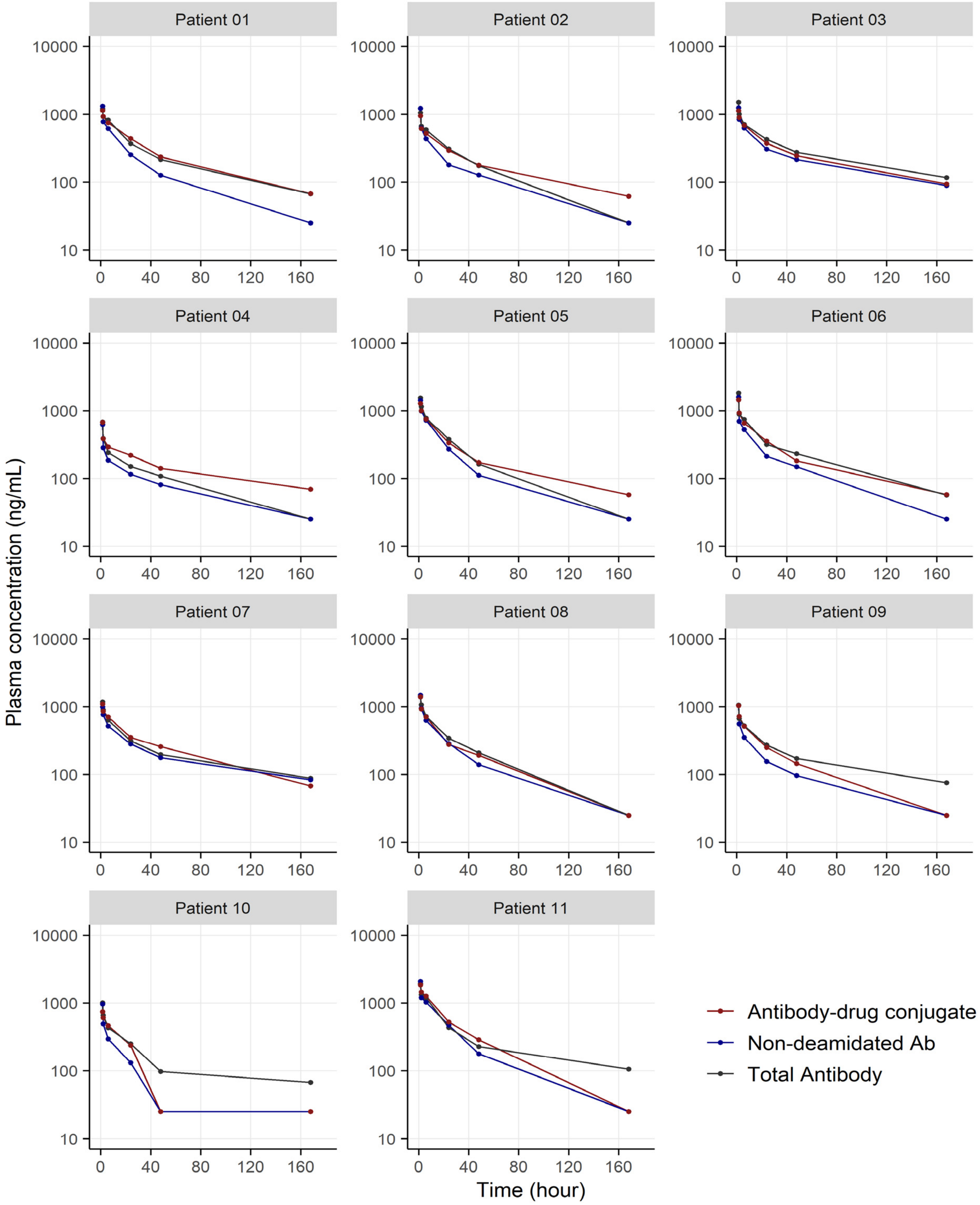

Supplement: Supplementary file 1 [file antibodies-12-00066-s001.zip › antibodies-2627395-supplementary.pdf]
